# An in-house *Kingella kingae* PCR in a large pediatric cohort: impact on diagnosis, antimicrobial stewardship, and clinical outcomes

**DOI:** 10.1128/jcm.00986-25

**Published:** 2025-10-09

**Authors:** Sophonie Jean Oyeniran, Amy L. Leber, Huanyu Wang

**Affiliations:** 1 Department of Pathology and Laboratory Medicine, Nationwide Children’s Hospital, Columbus, Ohio, USA; 2 Department of Pathology, The Ohio State University, College of Medicine 2647, Columbus, Ohio, USA; Endeavor Health, Evanston, Illinois, USA

**Keywords:** osteoarticular infection, pediatric, septic arthritis, Laboratory-developed PCR, Kingella kingae

## Abstract

*Kingella kingae* is a bacterial pathogen associated with bone and joint infections and a major pathogen in pediatric populations, particularly in those ≤5 years of age. Molecular detection methods have demonstrated significant increases in sensitivity compared to culture, allowing rapid and accurate identification. The objective of this study was to characterize the performance of an in-house *K. kingae* PCR (KKIN PCR) and determine its impact on antimicrobial utilization and clinical management in a pediatric population. Laboratory records from 1 September 2014 to 31 January 2024 were reviewed to identify subjects ≤18 years old who had KKIN PCR testing performed at Nationwide Children’s Hospital. Demographics, symptoms, radiologic and laboratory findings, hospitalization status, antimicrobial therapy, and clinical outcomes were analyzed. During the study period, 500 unique subjects with KKIN PCR ordered had complete data available, among whom 46 had *K. kingae* detected by PCR or culture. Compared to the clinical diagnosis, KKIN PCR had sensitivity, specificity, and positive and negative predictive values of 95.7, 100, 100, and 99.6%, respectively. Thirty-six (80%) subjects were detected by PCR alone, improving case identification by fourfold compared to culture (*n* = 9). Among subjects ≤5 years old, *K. kingae* was the most common organism detected. The median time to result for KKIN PCR was 26.5 h, and 38 (84%) subjects had antimicrobial modifications within 1 day of KKIN PCR report, resulting in de-escalation or antimicrobial optimization. This study demonstrates that routine use of in-house KKIN PCR significantly increased the diagnosis, facilitated timely and targeted treatment, and improved management of pediatric bone and joint infections.

**IMPORTANCE:**

*Kingella kingae* (KKIN) has long been recognized as a major cause of bone and joint infections in pre-school aged children. However, diagnosis and prevalence of KKIN infection are underestimated due to poor culture recovery. Previous studies have shown that molecular-based methods improve KKIN detection compared to culture, but these methods are not widely implemented or routinely used in clinical microbiology laboratories. This study describes the performance and clinical utility of an in-house laboratory-developed KKIN PCR in a large pediatric cohort over a nearly 10-year period. In addition to demonstrating improved KKIN detection compared to culture, it also shows that rapid availability of in-house KKIN PCR facilitated timely antimicrobial de-escalation and potentially contributed to shortened hospital length of stay compared to previous reports. This study highlights a critical diagnostic gap that can be alleviated with a validated laboratory-developed PCR to improve diagnosis and management of KKIN infections.

## INTRODUCTION

*Kingella kingae* is a bacterial pathogen associated with bone and joint infections. It is a major pathogen in pediatric populations, particularly in those less than 5 years of age. The organism is fastidious and can be difficult to isolate using conventional culture methods. While some improvement in isolation rates can be seen with the use of blood culture bottles for samples like joint fluid, the sensitivity is still relatively low, and there are no enhanced methods for the culture of alternate sample types, such as bone, bone aspirate, and other tissues (1). With the advent of PCR, multiple studies have demonstrated the increase in analytical sensitivity compared to culture (2). National clinical practice guidelines for acute bacterial arthritis and acute hematogenous osteomyelitis in pediatrics note that molecular testing for *Kingella kingae* may be beneficial in pre-school aged children with negative Gram stain and aerobic culture (3, 4). However, few studies have investigated the clinical impact of routine *K. kingae* PCR, particularly in a pediatric population.

The Clinical Microbiology and Immunoserology Laboratories at Nationwide Children’s Hospital (NCH) has offered an in-house *Kingella kingae* PCR (KKIN PCR) since 2014. In November 2022, clinical care pathways at NCH began recommending the use of dedicated *K. kingae* PCR testing in admitted children under 5 years of age with signs and symptoms of acute hematogenous osteomyelitis. The objective of this study was to characterize the performance of this PCR and determine the impact on antimicrobial utilization and clinical management in a pediatric population.

## MATERIALS AND METHODS

### Cohort selection and retrospective chart review

Records from NCH Clinical Microbiology and Immunoserology Laboratories were reviewed to identify subjects with *Kingella kingae* PCR testing performed from 1 September 2014 to 31 January 2024. Subjects ≤18 years of age were included. Orders for aerobic bacterial culture from blood (BC), joint/synovial fluid processed routinely (BFC) and in blood culture bottles (BFBC), and surgically collected aspirate and tissues (STC) for all subjects were reviewed. For subjects with *K. kingae* detected by culture or PCR, the electronic medical record was reviewed, where available, for demographics, symptoms, radiologic and laboratory findings, hospitalization status, antimicrobial therapy, and clinical outcomes as recorded in EMR by the treating clinician.

### Culture and susceptibility testing

BC were collected in aerobic (BacT/ALERT FA Plus) and anaerobic (BacT/ALERT SN) and incubated for up to 5 days using the BacT/ALERT 3D or Virtuo incubation systems (bioMérieux, Marcy L’Etoile, France). BFC specimens were centrifuged, and the sediment was plated to solid media (Sheep’s blood, chocolate and MacConkey agars) and thioglycolate broth for incubation up to 4 days. If more than 10 mL of fluid was received, aerobic and anaerobic blood culture bottles were inoculated with any remaining sediment and supernatant for incubation up to 5 days (BFBC). Specimens for STC were plated to solid media as above and thioglycolate broth for incubation up to 4 days. Recovered isolates were identified by Vitek2 or Vitek MS MALDI-ToF (bioMérieux, Marcy L’Etoile, France).

Antimicrobial susceptibility testing (AST) was performed on request for standard of care testing. Additionally, all available isolates were tested retrospectively for this study via Etest (bioMérieux, Marcy L’Etoile, France) with 5% sheep’s blood Mueller Hinton agar (Remel, Lenexa, KS, USA) or haemophilus test medium (Remel or Hardy Diagnostics, Santa Maria, CA, USA) and interpreted using CLSI M45 3rd edition (5). All isolates were evaluated for β-lactamase production via cefinase disk testing (BD BBL, Sparks, MD, USA) according to manufacturer’s instructions.

### *Kingella kingae* PCR

A real-time qualitative PCR assay targeting the major outer membrane protein gene (GenBank: KC142163.1) of *K. kingae* was developed for clinical use on joint fluid or tissue samples (KKIN PCR). Specificity of this assay was evaluated against other invasive bacterial pathogens (*Streptococcus pyogenes*, *Streptococcus agalactiae*, *Staphylococcus aureus*, Enterobacterales, and *Streptococcus pneumoniae*), normal cutaneous flora, viruses, human genomic DNA, as well as *K. dentitrificans* and *K. oralis*. In addition, *in silico* analysis was performed, and no cross-reactivity to other *Kingella* species was found, including the more recently described species *K. bonacorsii* and *K. negevensis*. Briefly, the specimen was extracted using the NucliSENS easyMag platform (bioMérieux, Durham, NC, USA). Five microliters of the eluate was added to a 25 µL total volume reaction mixture (1× TaqMan Universal Master Mix (Life Technologies, Grand Island, NY, USA), 0.9 µM of each primer (MOMP_F: 5′-TCGCAACGAAGTAGCTGTGTCT-3′ and MOMP_R: 5′-GCTGCAGATGCTTTCAAACG-3′), and 0.25 µM probe(MOMP_P: 5′-VIC-TGCATATAAAGTGAGCCCTG-MGB-3′). The amplification was carried out using the ABI 7500 thermocycler (Life Technologies, Grand Island, NY, USA) with the following running conditions: 50°C for 2 min, denaturation at 95°C for 10 min, and 45 cycles of 95°C for 15 s and 60°C for 1 min. The limits of detection are 400 CFU/mL for joint fluid and 500 CFU/mg for tissue. Clinical samples were batched and tested once daily.

### Time to result (TTR) calculation

For each subject, the time from sample collection (or order receipt for samples with add-on PCR requests placed >3 days from collection) to reported result of the first positive culture and/or positive PCR was determined. For blood cultures and body fluid in blood culture bottles, the time to Gram stain report following bottle positivity was utilized.

### Antibiotic impact

Antimicrobial therapy for subjects with *K. kingae* detected by culture or PCR was reviewed. Subjects were grouped by whether empiric antibiotic therapy was prescribed prior to culture or PCR result (empiric therapy or no empiric therapy) and whether empiric therapy was expected to be active (empiric KKIN active) or inactive (empiric KKIN inactive) against *K. kingae*.

### Data analysis

Data are reported as mean (range) or median (IQR). For comparisons of *K. kingae* detection between culture and PCR, data are presented as overall agreement, positive percent agreement (PPA), and negative percent agreement (NPA). Additionally, KKIN PCR performance was reported as sensitivity [95% CI], specificity [95% CI], positive predictive value (PPV) [95% CI], and negative predictive value (NPV) [95% CI] compared to final encounter diagnosis extracted from the hospital information system. For subjects with *K. kingae* detected by PCR or culture, *K. kingae* infection was confirmed via manual review of the EMR. For subjects with unspecified pyogenic arthritis, acute osteomyelitis, and/or discitis, the EMR was manually reviewed to determine whether the infection was clinically attributed to *K. kingae*. Contingency tables were analyzed by Chi-squared or Fisher’s exact test. Medians were compared using Mann-Whitney non-parametric tests. A *P*-value ≤ 0.05 indicates that the groups are significantly different. These analyses were performed with GraphPad Prism 10 (GraphPad Software, San Diego, CA, USA).

## RESULTS

### Cohort summary

**Fig 1:**
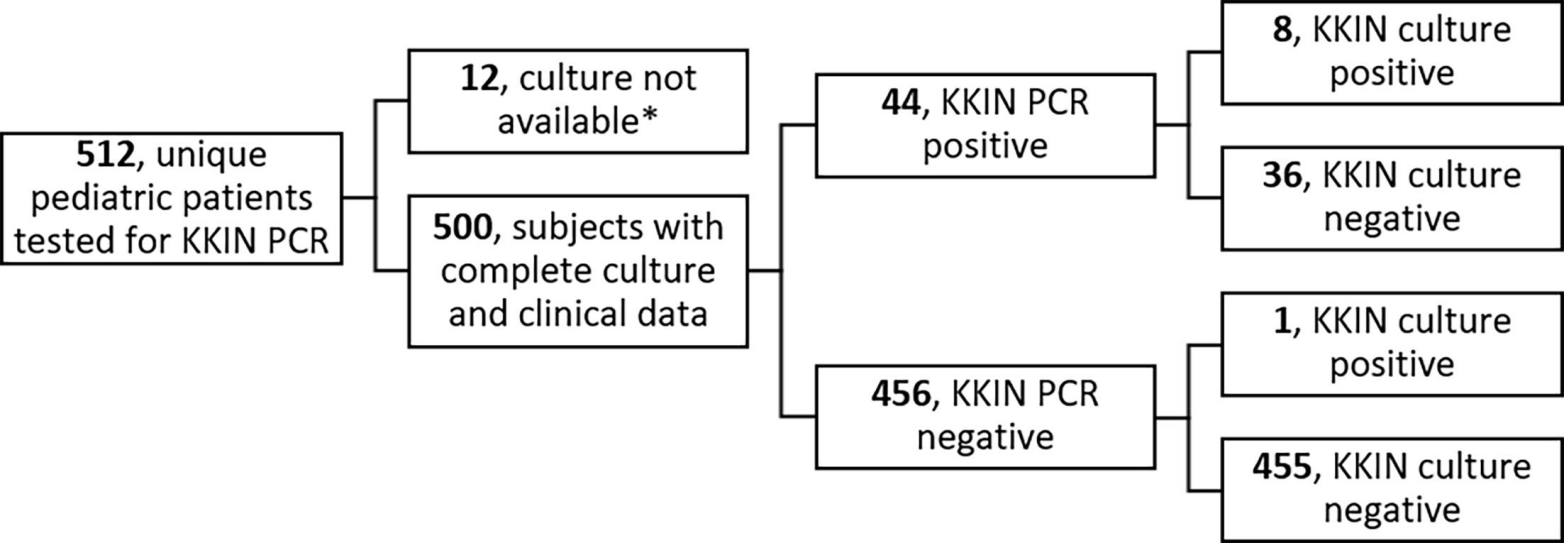
Flowchart of subjects tested for KKIN PCR. Study cohort of patients ≤ 18 years old tested by KKIN PCR between 1 September 2014 and 31 January 2024. KKIN PCR and culture (blood, body fluid, surgical tissue) results are shown. *Twelve subjects (2 KKIN PCR positive and 10 KKIN PCR negative) did not have culture results available and were excluded from further analysis.

From 1 September 2014 to 31 January 2024, 512 unique subjects had one or more joint fluid or tissue specimens tested with KKIN PCR. *K. kingae* was detected by PCR and/or culture from a total of 47 unique subjects (Fig. 1). Twelve subjects (two KKIN PCR positive, 10 KKIN PCR negative) did not have culture or clinical data available and were excluded from further analysis. Among 500 subjects with complete data available, 44 (8.8%) were KKIN PCR positive. One subject was PCR-negative but had *K. kingae* recovered from blood culture. In addition to the blood isolate above, culture recovered *K. kingae* from eight other subjects (one peripheral blood, one joint fluid, three joint fluid in blood culture bottles, and five surgical tissues). Demographic, clinical, and laboratory data for subjects with *K. kingae* detected are summarized in Table 1.

**TABLE 1:** Demographic, clinical, and laboratory summary of subjects with *K. kingae* detected by culture and/or KKIN PCR^*a*^

Characteristic	Value (no. of unique patients = 45)
Age median (IQR), months	17.6 (13.9–28.6)
Female, *n* (%)	24, 53%
Underlying conditions,^*b*^ *n* (%)	5, 11%
Presenting symptoms and labs	
Fever at admission	13, 29%
Any URTI symptoms	19, 42%
FARVPP-positive	6, 13.3%
CRP median, IQR (no. done)	2.9, 1.1–5.2 (43)
ESR median, IQR (no. done)	52, 34–76 (43)
WBC median, IQR (no. done)	11, 9.7–15 (43)
Body fluid nucleated cell count 10^4^, IQR (no. done)	7.7, 5.6–11.4 (22)
Body fluid diff % PMN, IQR (no. done)	90, 89–94 (22)
Diagnosis, *n* (%)	
ABA	23, 51%
AHO	15, 33%
ABA and AHO	3, 6.7%
Myositis	2, 4.4%
Spondylodiscitis/discitis	2, 4.4%
Hospital course, *n* (%)	
Admitted	44, 98%
Infectious diseases	39, 87%
Orthopedics	3, 6.6%
Other^*c*^	4, 8.9%
Surgical debridement or arthrocentesis, *n* (%)	43, 96%
LoS median (IQR), days	3 (1–17)

^*a*^ Abbreviations: URTI, upper respiratory tract infection; FARVPP, BioFire Film Array RP2/2.1 testing; CRP, C-reactive protein; ESR, erythrocyte sedimentation rate; WBC, white blood cell count; ABA, acute bacterial arthritis; AHO, acute hematogenous osteomyelitis; LoS, length of stay.

^*b*^ Underlying conditions: pre-term (2), CF carrier (1), galactosemia (1), and sickle cell trait (1).

^*c*^ Other: neurosurgery (2) and general medicine/surgery (2).

Subjects positive for *K. kingae* had a median (IQR) age of 17.6 months (13.9–28.6), and 53% were female. Most subjects (90%) had no underlying conditions. Less than 30% of subjects presented with fever at admission, and most had normal to mild elevations in inflammatory markers (C-reactive protein, erythrocyte sedimentation rate, and white blood cell count, Table 1). Nearly all subjects were admitted (98%) primarily to Infectious Disease and Orthopedics units (87 and 6.7%, respectively) for a median (IQR) of 3 (1–17) days. Forty-three (96%) subjects had surgical interventions, including arthrocentesis, arthrotomy, incision and drainage, IR/CT, and/or US-guided aspiration, and bone biopsy. Across all 45 subjects, 23 (51%) were diagnosed with acute bacterial arthritis (ABA), 15 (33%) with acute hematogenous osteomyelitis (AHO), three (6.7%) with ABA and AHO, two (4.4%) with myositis, and two (4.4%) with (spondylo)discitis.

### Performance of KKIN PCR

Results from the joint fluid and tissue KKIN PCR were compared to BC, BFC, BFBC, and STC cultures per subject (*n* = 500). Subjects were considered KKIN PCR-positive if at least one sample tested positive by PCR or culture-positive if *K. kingae* was recovered from at least one sample. The PPA, NPA, and total agreement between KKIN PCR and culture were 88.9, 92.7, and 92.6%, respectively (Table 2). KKIN PCR testing was batched and tested daily during the study period, and *K. kingae* PCR detections had significantly faster median TTR compared to positive culture (26.5 h vs. 47 h (*P*-value = 0.03, Fig. S1). There was no difference in the KKIN PCR geometric mean C_T_ values of subjects whether or not *K. kingae* was recovered in culture (30.9 vs. 31.9, *P*-value = 0.39). All subjects with positive KKIN PCR and negative culture (*n* = 36), as well as positive culture and negative KKIN PCR (*n* = 1), had a clinical diagnosis of *K. kingae* infection and received *K. kingae*-directed antimicrobial therapy. Upon review of the final clinical diagnosis, one additional subject that was both culture- and KKIN PCR-negative had a clinical diagnosis of *K. kingae* infection based on broad-range PCR detection from a distinct sample. Overall, compared to the clinical diagnosis, KKIN PCR had sensitivity, specificity, positive and negative predictive values [95% CI] of 95.7% [85.5–99.2], 100% [99.2–100], 100% [92–100], 99.6% [98.4–99.9], respectively (Table 2).

**TABLE 2:** Performance of KKIN PCR compared to culture^*a*^

	KKIN culture-positive	KKIN culture-negative
KKIN PCR vs. culture^*b*^		
KKIN PCR-positive	8	36
KKIN PCR-negative	1	455
Overall agreement	92.6% (463/500)	
Positive percent agreement	88.9% (8/9)	
Negative percent agreement	92.7% (455/491)	

	Clinical*—K. kingae* infection	Clinical—NOT *K. kingae* infection
KKIN PCR vs. clinical diagnosis^*b*^		
KKIN PCR-positive	44	0
KKIN PCR-negative	2	454
Sensitivity [95% CI]	95.7 [85.5–99.2]	
Specificity [95% CI]	100% [99.2–100]	
PPV [95% CI]	100% [92–100]	
NPV [95% CI]	99.6% [98.4–99.9]	

^*a*^ Analysis excludes 12 subjects with no culture or clinical data available.

^*b*^ Fisher’s exact test *P*-value < 0.0001.

### *K. kingae* culture recovery and susceptibility profile

Among subjects with *K. kingae* detected by culture and/or KKIN PCR, most had blood cultures collected (82%, 37/45) of which two (5.4%) had *K. kingae* recovered. Both flagged positive within 48 h of collection.

Excluding blood cultures, *K. kingae* positive subjects had, on average (range), two (1–4) specimens submitted for culture. Twenty-nine (64%) subjects had ≥1 joint fluid specimens (BFC) submitted. Thirteen subjects (13/29, 45%) had sufficient specimen for processing with blood culture bottles (BFBC). One subject had *K. kingae* recovered from both routinely processed joint fluid (BFC) and joint fluid in blood culture bottles (BFBC); two additional subjects had *K. kingae* recovered from only BFBCs. Thirty-five (78%) subjects had surgically collected abscesses/aspirates, tissue, and/or bone submitted for culture (STC), of which five (14%) had *K. kingae* isolated. Overall, across all specimen types, *K. kingae* was recovered by culture in 9/45 (20%) subjects, 11 total specimens.

*K. kingae* susceptibility testing was performed as part of the standard of care for 67% (6/9) culture-positive subjects. Additionally, all available isolates (*n* = 6) were tested retrospectively for this study. All were uniformly susceptible to trimethoprim-sulfamethoxazole, meropenem, ciprofloxacin, levofloxacin, ampicillin, amoxicillin-clavulanate, penicillin, and azithromycin and found to be β-lactamase negative.

### Pathogen distribution of subjects tested by KKIN PCR

**Fig 2:**
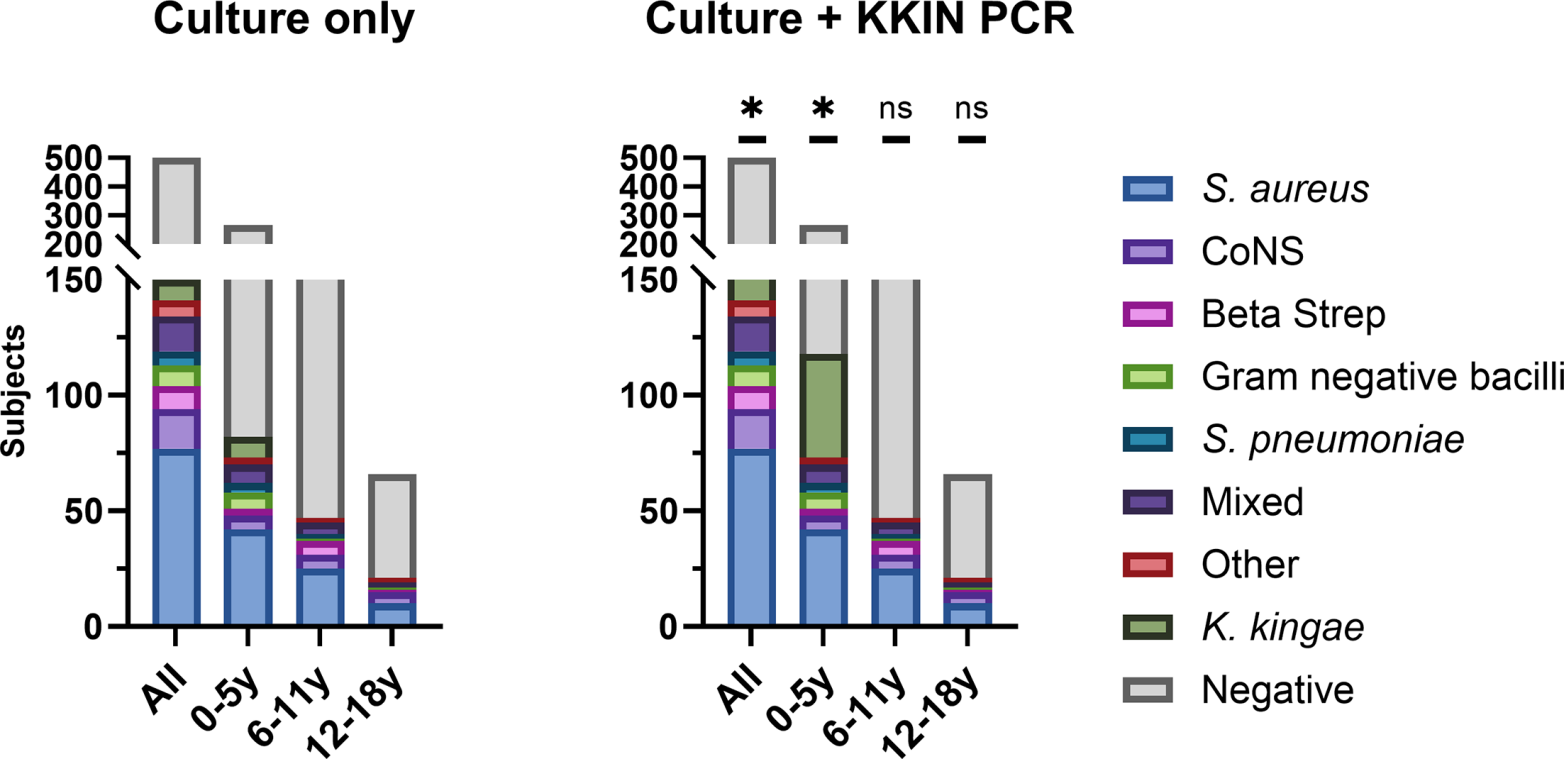
Pathogen distribution of all subjects tested by KKIN PCR. Organisms detected by culture only or culture and KKIN PCR are shown across all subjects (*n* = 500), subjects 0–5 years old (*n* = 267), 6–11 years old (*n* = 167), and 12–18 years old (*n* = 66). Chi-squared analysis of pathogen distribution with culture only and culture + KKIN PCR across all age groups is shown; * denotes *P*-value ≤ 0.001, and ns indicates not statistically significant. Culture results from all subjects are further described as follows: 77 *S. aureus*, 17 coagulase-negative staphylococci (CoNS), 10 beta-hemolytic streptococci (Beta Strep: seven *Streptococcus pyogenes* [GAS], two *Streptococcus agalactiae* [GBS], one *Streptococcus canis/dysgalacticae* [Group C/G]); 9 gram-negative bacilli: two *Enterobacterales*, three *Salmonella* spp., two *Pseudomonas aeruginosa*, two *Haemophilus influenzae*, one *Moraxella* spp.; mixed: four *Staphylococcus aureus* + other gram-positive bacteria, three gram-positive and -negative bacteria, six mixed gram-positive bacteria, two normal flora; and other: two viridans group streptococci, two *Bacillus* spp., two *Staphylococcus lugdunensis*, and one *Mycobacteria goodii*.

To better estimate the prevalence of *K. kingae* relative to other etiological agents of culture-confirmed pediatric bone and joint infections, culture data for all subjects with data available (*n* = 500) tested for *K. kingae* by PCR were reviewed. While most cultures were negative, 28% (141/500) of subjects were culture positive with organisms other than *K. kingae*. Across all subjects, *Staphylococcus aureus* (*n* = 77, 15%) was the most common bacterium recovered, followed by coagulase-negative staphylococci (*n* = 17, 3.4%), beta-hemolytic streptococci (*n* = 10, 2%: *Streptococcus pyogenes* [GAS]—seven, *Streptococcus agalactiae* [GBS]—two, *Streptococcus canis/dysgalacticae* [Group C/G]—one), gram-negative bacilli (*n* = 9, 1.8%), and *Streptococcus pneumoniae* (*n* = 6, 1.2%) (Fig. 2). Among subjects 0–5 years of age, *K. kingae* was the most common organism detected, surpassing *S. aureus* (47/267 vs. 42/267) when both culture and KKIN PCR results were considered (Fig. 2).

### Antimicrobial management and outcomes

**Fig 3:**
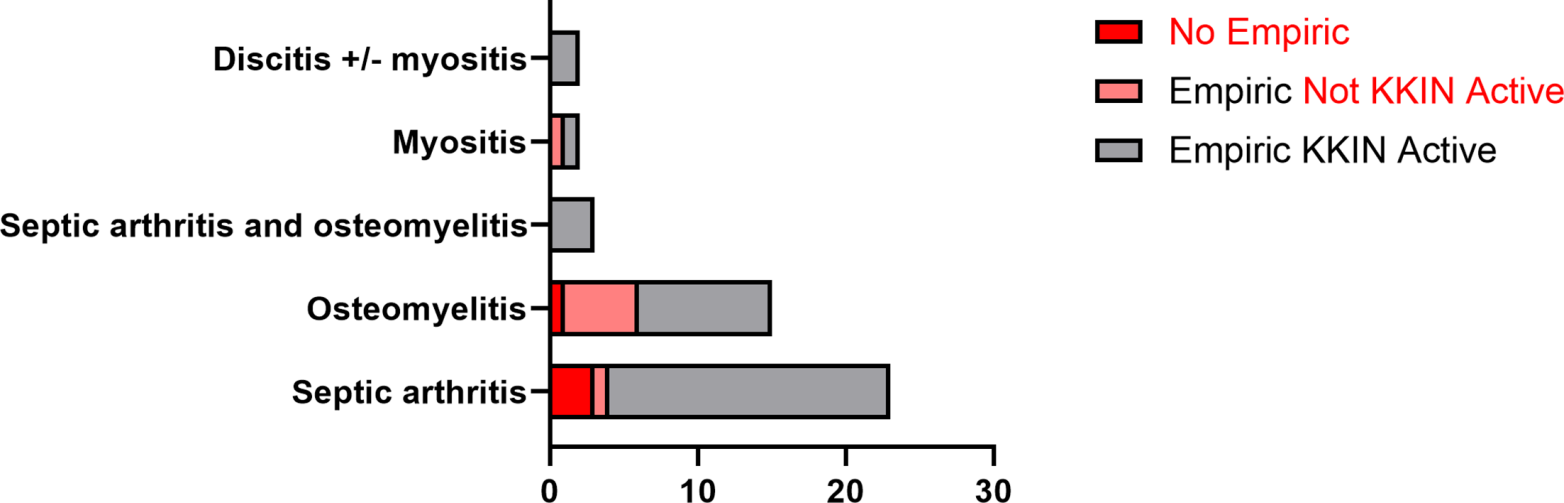
Empiric antibiotic coverage by clinical diagnosis among KKIN-positive patients. The bar plot depicts the number of subjects with septic arthritis/ ABA (*n* = 23), osteomyelitis/ AHO (*n* = 15), ABA and AHO (*n* = 3), myositis (*n* = 2), and spondylodiscitis (*n* = 2) who were treated empirically with an antimicrobial regimen expected to have activity against *K. kingae* (gray), no activity against *K. kingae* (pink), or who received no empiric antimicrobial therapy prior to reporting of KKIN PCR/culture (red).

Antimicrobial therapy was reviewed for all 45 subjects with *K. kingae* detected and data available. Eleven subjects received no empiric therapy (*n* = 4, 8.9%) or empiric therapy that was not active against *K. kingae* (*n* = 7, 15.6%), among which eight had *K. kingae* detected by PCR only. Seven of these eight subjects were diagnosed with osteomyelitis or myositis (Fig. 3). All of these 11 subjects received targeted antimicrobials following KKIN PCR detection. Thirty-four (75.6%) subjects received antimicrobials active against *K. kingae* empirically (Table S1), of which 32 had further optimization of antimicrobials following KKIN PCR detection. (29/32) of antimicrobial modifications in this group occurred on the same day KKIN PCR was reported.

Across all subjects, KKIN PCR results made a direct impact on patient management in 93% (42/45) subjects, with antimicrobial changes and hospital discharge occurring on the same day of the KKIN PCR report in 84% (38/45) and 58% (26/45) subjects, respectively. Most antimicrobial modifications were de-escalations, as 62% (28/45) of the subjects were on combination therapy with broad-spectrum agents empirically, while no subjects remained on multiple agents, and only one subject remained on a broad-spectrum agent for targeted therapy (Table S1).

Subjects were treated for a median duration of 21 days (IQR; range: 21–28; 18–196). There was no difference in duration of therapy among subjects with *K. kingae* detection by culture versus PCR only (data not shown). Follow-up medical encounters were documented for 44/45 KKIN positive subjects, at a median (IQR) of 28 (18–78) days after discharge or sample collection for subjects not admitted. Despite the lack of susceptibility data for those subjects that were positive by only KKIN PCR (*n* = 36), all subjects were successfully treated with complete resolution of symptoms on follow-up and no evidence of treatment failure.

## DISCUSSION

KKIN is an important pathogen particularly in pediatric patients. Previous studies primarily from Europe have demonstrated that use of molecular methods, including targeted KKIN PCR or broad-range sequencing assays, significantly improved the detection of osteoarticular infections due to *K. kingae* (6–11). In this study, we evaluated the use of an in-house KKIN PCR in 500 pediatric subjects over a nearly 10-year period. KKIN PCR detected *K. kingae* in 44 subjects and outperformed culture. Most subjects with *K. kingae* infection were detected by PCR alone (80.0%, 36/45), improving case identification by greater than fourfold compared to culture (culture: 9 versus PCR: 44). Notably, while we show that KKIN PCR significantly improved the diagnosis of *K. kingae* infection in this cohort, KKIN PCR of joint fluid missed one subject with blood culture-confirmed *K. kingae* joint infection. It is unclear whether this was due to undetectable levels of *K. kingae* in the joint fluid sample, which was also culture-negative, or error during clinical testing. KKIN PCR of a blood sample from this subject may have detected *K. kingae*; however, blood samples were not systematically tested by KKIN PCR during the study period. In one report of 31 children with *K. kingae* arthritis, the authors found that of 15 subjects with peripheral blood tested by KKIN PCR, none had *K. kingae* detected. This suggests that the bacterial load of *K. kingae* in blood during an infection may be low and blood may not be an appropriate specimen type for detecting *K. kingae* acute bacterial arthritis (12).

Our findings also confirm previous work identifying *K. kingae* as the leading cause of osteoarticular infection in pre-school aged children when molecular methods are used (13, 14). All *K. kingae* detections in our study were from subjects 5 years old or younger despite the fact that 46% of subjects tested by KKIN PCR were aged 6 years or older. Using only culture-based methods, *K. kingae* was recovered from only 1.8% (9/500) subjects in the entire cohort and 3.4% (9/267) of subjects 5 years old or younger. However, when KKIN PCR positive detections were included, the pathogen distribution was significantly different with *K. kingae* detected in 9% (45/500) of the entire cohort and 16.8% (45/267) of all children ≤5 years old. Additionally, 38% (45/118) of children ≤5 years old with a pathogen identified had *K. kingae* osteoarticular infection, surpassing *S. aureus* (35%; 42/118) in this age group. A recent multicenter study of two pediatric institutions in the Southeastern US retrospectively reviewed 453 pediatric subjects with musculoskeletal infections over a 9-year study period and identified *K. kingae* as the etiological agent in only 1% infections (15). The authors acknowledged that the low *K. kingae* prevalence reported in this study was likely attributed to the low rates of molecular testing at both study sites.

Despite the high prevalence of *K. kingae* in pediatric OAI, many US clinical laboratories do not offer *K. kingae* molecular testing in-house (9). This may be due in part to cost justification of a relatively low-volume test and also the dearth of commercial *in vitro* diagnostics (IVDs) targeting *K. kingae*. To date, the only commercial IVD with regulatory approval in the US that detects *K. kingae* directly is the BioFire Joint Infection Panel (BF-JIP, BioMérieux, Salt Lake City, UT, USA). This IVD also detects 30 other potential pathogens and eight antimicrobial resistance genes common in ABA and prosthetic joint infection from synovial fluid in approximately 1 h. Although this test has been successfully used to diagnose ABA due to *K. kingae* in children (16–18), it is better suited for the complex and often polymicrobial prosthetic joint infections typically seen in adult patients. Additionally, the BF-JIP is not approved for use with tissue or biopsy specimens, potentially limiting its utility in patients with osteomyelitis, myositis, and (spondylo)discitis, particularly if there is no contiguous joint infection. In our cohort, 19 (40%) subjects had osteomyelitis, myositis, and/or discitis, of whom only seven had joint fluid specimens submitted for culture and KKIN PCR. The BF-JIP would have had no diagnostic utility in these 12 subjects in our cohort, unless an LDT validation of bone and tissue specimens for use with the BF-JIP was performed and acceptable.

Some US clinical laboratories may rely on stand-alone *K. kingae* PCR or broad-range bacterial sequencing via send-out testing performed at reference laboratories in the absence of in-house KKIN PCR testing (19). More recently, plasma microbial cell-free DNA sequencing (Karius, Redwood City, CA) has been used to identify infections due to *K. kingae* in pediatric patients. Ahmed and colleagues reported *K. kingae* detections by Karius in 10 pediatric subjects with vertebral infections. Karius facilitated the diagnosis without the need for an invasive surgical procedure in eight subjects. However, four (40%) subjects also had Karius co-detections of other oropharyngeal microorganisms, complicating interpretation of the result and necessitating either tissue-specific detection and/or careful review of clinical, epidemiologic, and laboratory findings to confirm the diagnosis (20). In our cohort, seven subjects with *K. kingae* PCR or culture detection had broad-range sequencing (*n* = 6) or Karius (*n* = 1) testing submitted to a reference laboratory. Four (66.7%, 4/6) samples submitted for broad-range sequencing were negative, highlighting the difference in sensitivity between the targeted PCR and broad-range sequencing and the potential risk of missed detection when relying solely on broad-range sequencing.

Antimicrobial therapy for *K. kingae* bone and joint infections can be successfully transitioned to a narrow-spectrum, oral antibiotic with evidence of clinical improvement (4). Therefore, timely identification of this specific pathogen can potentially reduce the duration of broad-spectrum antimicrobial therapy and length of hospitalization, particularly in subjects with acute bacterial arthritis without osteomyelitis. In our cohort, send-out broad-range bacterial/16S rDNA PCR or Karius testing took a median (IQR) of 7.8 days (7.1–10.2), significantly longer than in-house testing (median, IQR: 1.2 days, 0.3–2.2, *P*-value < 0.0001). Given that 84% of antimicrobial modifications were made on the same day of the KKIN PCR results, it is possible that antibiotic de-escalation and/or optimization would have been delayed in our cohort without an in-house KKIN PCR test. Additionally, 58% (26/45) of subjects in our cohort were discharged on the same day of the KKIN PCR report with a median (IQR) length of stay (LoS) of 3 days (1–17). In one study of 40 children with *K. kingae* ABA in Texas, Villani and colleagues reported an average LoS of 3.58 days and demonstrated that the LoS of children with *K. kingae* ABA was significantly shorter than that of children with septic arthritis due to another pathogen (5.53 days) or when no pathogen was identified (4.27 days) (9). In our study, among subjects with *K. kingae* ABA (*n* = 23), the median (IQR) LoS was 2 days (2–3). Twenty-one (91%) subjects with ABA had antimicrobial modifications made on the same day of the KKIN PCR report, resulting in transition to oral antibiotics and hospital discharge in 14 (68%, 14/21) subjects within 1 day of the KKIN PCR report. While the reported inflammatory markers (CRP, ESR, WBC, joint fluid cell count, and % neutrophils) in Villani et al.’s study were comparable to this report, we observed an LoS of 1–2 days shorter. One plausible explanation is that in their study, *K. kingae* was detected via send-out broad-range or targeted PCR while our testing was performed in-house.

A limitation of molecular-based *K. kingae* detection may be the lack of susceptibility data to guide targeted treatment. However*, K. kingae* is generally susceptible to β-lactams; as such, they are often the first line drugs to treat *K. kingae* BJIs. Beta-lactamase detection and resistance to penicillin have been reported, but these isolates remained susceptible to cephalosporins (21, 22). Although susceptibility testing on recovered isolates may be useful to inform susceptibility trends, routine testing may not be needed for patient management. In our study, despite the fact that the majority of the subjects were only positive by KKIN PCR and did not have antimicrobial susceptibility results to guide antimicrobial therapy, all patients were treated successfully. This is consistent with previous reports that children with *K. kingae* BJIs often had favorable clinical outcomes following appropriate antibiotic treatment (23, 24).

There are limitations to our study. First, the pathogen distribution reported for the subjects in this study was limited to bacterial culture-proven or KKIN PCR-positive and so does not account for Lyme arthritis or other atypical causes of osteoarticular infection in children. Also, while it is possible that molecular testing targeting the other bacterial pathogens reported may have further increased their prevalence relative to *K. kingae*, our rates of other bacterial etiologies are consistent with other microbiologic descriptions of pediatric OAI (9, 15).

To conclude, in this large North American pediatric cohort, we report that routine use of in-house *K. kingae* PCR significantly increased the diagnosis of *K. kingae* osteoarticular infections compared to optimized culture methods. This study confirms the high prevalence, particularly in children less than 5 years of age, and clinical presentation of *K. kingae* bone and joint infection in a cohort of over 500 pediatric subjects and reinforces the need for molecular-based testing for diagnosis of *K. kingae* infections routinely. The rapid availability of in-house KKIN PCR testing in our study facilitated antimicrobial optimization and hospital discharge on the same day of the KKIN PCR report in 84% and 58% subjects, respectively. This potentially contributed to the shortened hospital length of stay observed among subjects with ABA compared to previous reports. The lack of commercial single-plex assays with regulatory approval for detection of *K. kingae* in clinical samples and the extended turn-around time of send-out testing highlight an example of a critical diagnostic gap that can be readily alleviated with a validated laboratory-developed PCR-based testing for *K. kingae*.

## SUPPLEMENTAL MATERIAL

Supplemental material jcm.00986-25-s0001.docx Empiric and targeted antimicrobial regimens of the study cohort; time to culture and PCR result in subjects with *K. kingae* detected.

ASM does not own the copyrights to Supplemental Material that may be linked to, or accessed through, an article. The authors have granted ASM a non-exclusive, world-wide license to publish the Supplemental Material files. Please contact the corresponding author directly for reuse.
