# Supplementary material for: An in-house Kingella kingae PCR in a large pediatric cohort: impact on diagnosis, antimicrobial stewardship, and clinical outcomes
Source: J Clin Microbiol. 2025 Oct 9;63(11):e00986-25. doi: 10.1128/jcm.00986-25 (PMC12607887; doi:10.1128/jcm.00986-25)
Supplement: Supplemental material — Empiric and targeted antimicrobial regimens of the study cohort; time to culture and PCR result in subjects with K. kingae detected. [file jcm.00986-25-s0001.docx]

**Title:** An in-house *Kingella kingae* PCR in a Large Pediatric Cohort: Impact on Diagnosis, Antimicrobial Stewardship and Clinical Outcomes

**Authors:** Sophonie Jean Oyeniran^1, 2^, Amy L. Leber^1, 2^, and Huanyu Wang*^1, 2^

**Affiliations:**

^1^Department of Pathology and Laboratory Medicine, Nationwide Children’s Hospital, Columbus, OH 43205

^2^Department of Pathology, The Ohio State University, College of Medicine, Columbus, OH 43203

^*^Corresponding author: HW, hunayu.wang@nationwidechildrens.org

ALL and HW contributed equally to this article

**Supplemental Tables and Figures**

**Table S1. Empiric and targeted antimicrobial regimens of study cohort**

| **Antimicrobial regimen** | **% (n)** |  |  |
| --- | --- | --- | --- |
| **Empiric therapy (before KKIN PCR/culture result)** | **N=45** | **Antimicrobial spectrum of activity** |  |
| Cefazolin | 4.4% (2) | T |  |
| Cephalexin | 2.2% (1) | T |  |
| Cefazolin + clindamycin | 8.9% (4) | C |  |
| Cefazolin + vancomycin | 4.4% (2) | C/B |  |
| Cefepime + clindamycin | 2.2% (1) | C/B |  |
| Ceftriaxone + clindamycin | 38% (17) | C/B |  |
| Ceftriaxone + vancomycin | 11% (5) | C/B |  |
| Ciprofloxacin + clindamycin | 4.4% (2) | C/B |  |
| Clindamycin | 15% (7) | I |  |
| None | 8.9% (4) | I |  |
| **Targeted therapy (after KKIN PCR/culture result)** | **N=45** |  |  |
| Cephalexin | 64% (29) | T |  |
| Amoxicillin-clavulanate | 18% (8) | T |  |
| Cefdinir | 8.9% (4) | T |  |
| Cefazolin | 4.4% (2) | T |  |
| Ceftriaxone | 2.2% (1) | B |  |
| Cefuroxime | 2.2% (1) | T |  |
| **Abbreviations:** I—Ineffective or no empiric therapy; T—targeted therapy; C—combination therapy; B—broad-spectrum therapy. | | | |

**Figure S1. Time to culture and PCR result in subjects with *K. kingae* detected**

**
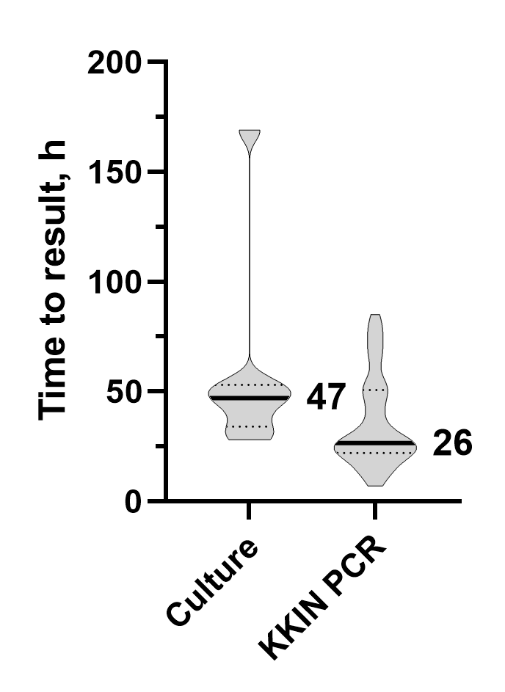
**

**Figure S1. Time to culture and PCR result in subjects with *K. kingae* detected.** Violin plot showing median (solid, black) and first and 1^st^ and 3^rd^ quartiles (dashed, grey) time to result for first positive culture (n=9) or PCR (n= 44) per subject. Positive KKIN PCR had faster median time to result than positive culture (Mann-Whitney test, *P*=0.03).
